# Correlation Analyses on Physical and Mechanical Parameters of Concrete in Marine Environments

**DOI:** 10.3390/ma15051812

**Published:** 2022-02-28

**Authors:** Jinwei Yao, Hui Song, Yizhan Yang

**Affiliations:** 1 Zhejiang Business Technology Institute, Ningbo 315012, China; 2 Key Laboratory of Impact and Safety Engineering, Ministry of Education, School of Mechanical Engineering and Mechanics, Ningbo University, Ningbo 315211, China; 3 Jiangxi Provincial Key Laboratory of Hydraulic & Civil Engineering Infrastructure Security, Nanchang Institute of Technology, Nanchang 330099, China; songhui@nit.edu.cn; 4 Hubei Key Laboratory of Engineering Structural Analysis and Safety Assessment, Department of Mechanics, School of Aerospace Engineering, Huazhong University of Science and Technology, Wuhan 430074, China; yzyang1993@hust.edu.cn

**Keywords:** marine environment, corrosion, correlation, macro-parameter, micro-analysis

## Abstract

This work explored the correlation among the physical and mechanical parameters of concrete in marine environments during corrosion. Concrete materials with varied water–cement ratios (*w*/*c*) were soaked in four kinds of simulated seawater, including a compound solution of sodium chloride (NaCl) and magnesium sulfate (MgSO_4_), a solution of MgSO_4_, a solution of sodium sulfate (Na_2_SO_4_)_,_ and clear water. The macroscopic physical and mechanical parameters, such as compressive strength, tensile strength, mass change and ultrasonic wave velocity, were measured. The damage mechanism of corrosion products and microstructures was analyzed using microscopic approaches including scanning electron microscopy (SEM), X-ray diffraction (XRD), etc. Linear correlation was carried out on the abovementioned physical parameters, and the results revealed that, during corrosion, the correlation between compressive and tensile strength and mass (excluding Na_2_SO_4_ solution) is positive (mostly highly correlated), while that with wave velocity is poor (mostly moderately correlated).

## 1. Introduction

Study of the corrosion of concrete in the marine environment has always been a topic of focus [1,2,3,4,5]. Factors include the corrosion of reinforcements in concrete, the physical and chemical erosion caused by harmful salt ions, and freeze–thaw cycles that can drive corrosion and deterioration [6,7,8,9,10,11,12]. As a multi-salt solution, seawater consists of chloride ions, sulfate ions, and magnesium ions, which corrode concrete and reduce its service life. Coastal waters abound in such harmful ions. Table 1 shows the content of major ions in the seawater of China’s coastal ports [13]. It can be seen from Table 1 that the content of chloride ions (Cl^−^) in seawater in the various places is higher than that of sulfate ions (SO_4_^2^^−^), and the content of sulfate ions is in turn higher than that of magnesium ions (Mg^2+^). The three ions relatively abound in the sea areas near Dalian, Tianjin, Qingdao, and Xiamen; the sulfate ions and magnesium ions are rich in Lianyungang; while the sulfate ions and magnesium ions are the least observed in seawater near Beilun. The corrosion pressure of marine materials with high content of harmful ions is relatively high.

The scholarly literature is rich in studies on the physical and mechanical properties of concrete materials in marine environments, with some studies regarding the damage evolution of concrete [14,15,16,17,18,19,20]. According to Dehwah [14], no apparent damage was observed in concrete samples soaked in a compound solution of sodium sulfate (Na_2_SO_4_) and sodium chloride (NaCl); by contrast, serious deterioration was found in samples treated with a compound solution of magnesium sulfate (MgSO_4_) and sodium chloride (NaCl). Niu et al. [15] elucidated the physical and mechanical properties of concrete materials, including visual change, relative dynamic elasticity modulus, weight loss, compressive strength and damage thickness, in three kinds of multi-salt corrosion environments, namely Na_2_SO_4_ solution, MgSO_4_ solution and a compound solution of Na_2_SO_4_ and NaCl, and observed that deterioration in the MgSO_4_ solution was worse than that in other sulfate solutions. Jin et al. [16] compared two groups of concrete corroded by sulfate and chloride solution, respectively, and proposed three stages of relative dynamic elasticity modulus for concrete soaked in sulfate: a linearly increasing stage, a stable stage, and a declining stage. The presence of corrosive ions, including Cl^−^, SO_4_^2^^−^, and Mg^2+^, in seawater is critical for chemical corrosion of and damage to concrete materials, and various ions can interact with each other to promote or inhibit corrosion [21,22,23,24,25,26,27].

There exist certain correlations among the physical and mechanical parameters of concrete in marine environments subjected to corrosion, which are often universally related, interdependent and mutually constrained. However, correlation analysis on the physical parameters of concrete in a corrosion environment is rare. Harbulakova et al. [28] investigated the effects of three types of concrete corrosion (acidic, sulfate and leach) and found a correlation between concentration of leaching silica and calcium and pH. Barbudo et al. [29] revealed the limited gypsum amount of recycled aggregate was 4.4%, based on correlation analysis between solubility and leachability. All these studies support further research on correlations among the physical properties of concrete materials corroded by multi-salt solutions.

This paper studied multi-salt corrosion of concrete samples (both ordinary concrete and high-strength concrete) in simulated seawater. The samples’ physical and mechanical properties were analyzed based on tests of compressive and tensile strength, mass loss and ultrasonic wave velocity during corrosion; the roles played by corrosion solutions in the samples’ microscopic damage mechanisms were compared by measuring their microscopic composition. The principal objective of this paper was to analyze the internal correlation of each physical parameter of concrete in a corrosive environment through correlation analysis of strength, mass and wave velocity changes, and to reveal the internal relationship. The results provide a new way to characterize the damage to concrete materials in corrosive environments through the correlation of various physical and mechanical parameters.

## 2. Experimental Methods

### 2.1. Materials and Instrumentation

**Figure 1:**
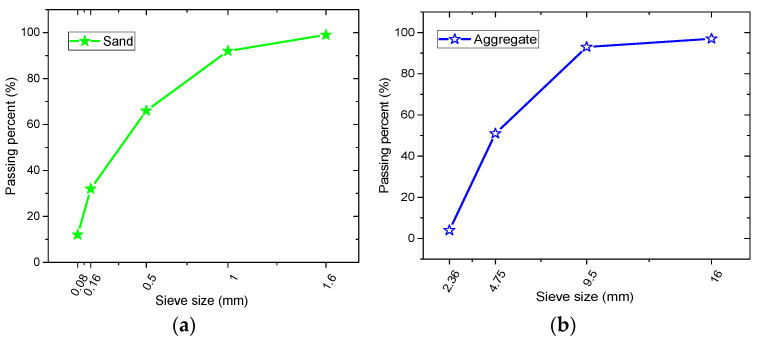
Particle size distributions: (**a**) sand, (**b**) aggregate.

Portland cement categorized as P·O 42.5 was utilized; its chemical composition is shown in Table 2. The fine aggregate was composed of Chinese ISO standard sand [30] (particle distribution shown in Figure 1a), while the coarse aggregate was 5~16 mm continuous graded macadam (particle gradation shown in Figure 1b). The corrosive salt solutions were a mixture of water and salts including Na_2_SO_4_, MgSO_4_, and NaCl.

### 2.2. Mix Design for Concrete and Specimen Preparation

The concrete samples employed were high-strength concrete (*w*/*c* = 0.33) and ordinary concrete (*w*/*c* = 0.50). They also vary in size: the former is a cube (100 mm × 100 mm × 100 mm) for compressive strength testing and the latter a cylinder (100 mm in diameter and 50 mm in thickness) for tensile strength testing. The casting samples were demolded after 24 h natural curing. Afterwards, the samples were soaked in tap water for curing for 28 days, and then immersed in solutions for immersion corrosion testing to simulate the actual marine environment. To ensure consistency of solution concentration, plastic film was used to cover the immersion box, thus preventing moisture volatilization, and the solution was refreshed every other month.

The four corrosion solutions used were as follows: 5% MgSO_4_ + 10% NaCl solution (MC), 5% MgSO_4_ solution (MS), 5% Na_2_SO_4_ solution (SS) and clear water (CW, control solution). Their concentrations are shown in Table 3. Concentrations of corrosive solutions were selected with reference to ASTM C1012/C1012M-2015 [31] and GB/T50082-2009 [32].

### 2.3. Measurement

The immersion corrosion test lasted for 20 months, and samples were taken every month for the first year, followed by the 15th and 20th months, to test physical and mechanical parameters including compressive strength, tensile strength, mass and ultrasonic wave velocity. In addition, microscopic corrosion composition testing was conducted in the 10th month.

#### 2.3.1. Compressive and Tensile Strength Testing

**Figure 2:**
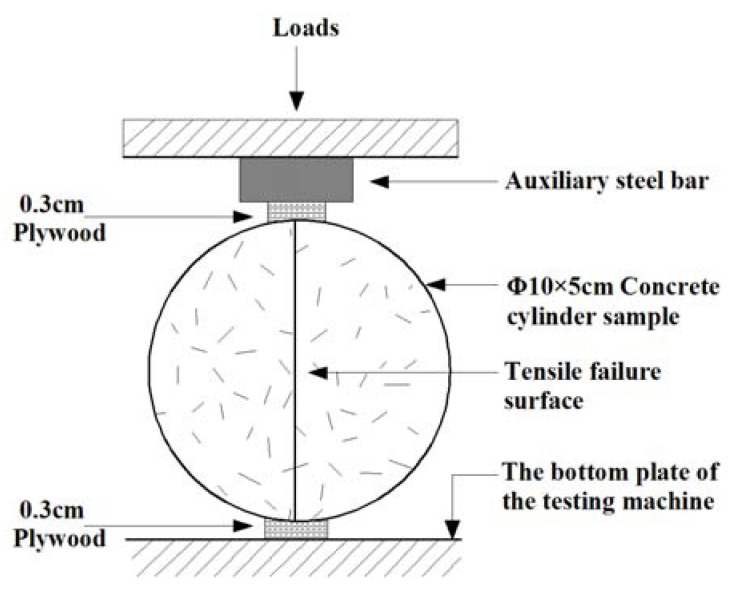
Schematic diagram of splitting tensile test method.

**Figure 3:**
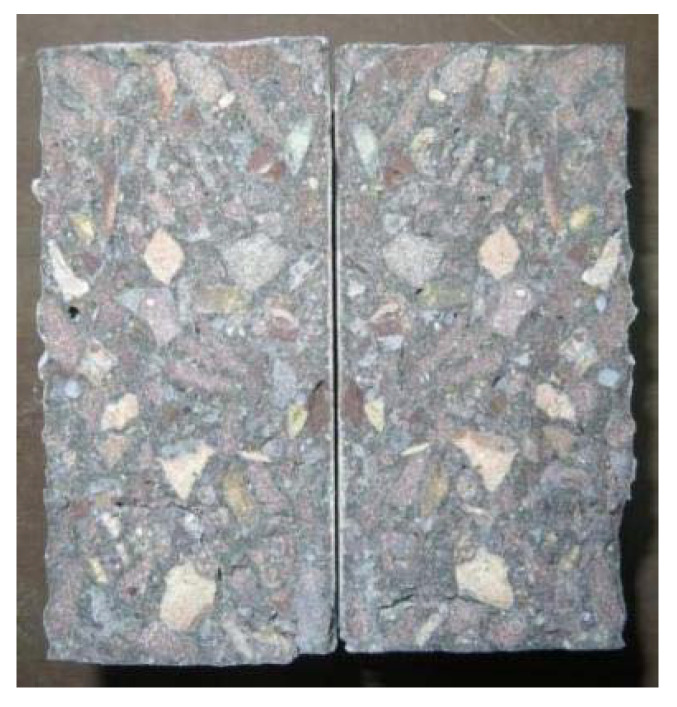
Fracture surface of tensile test piece.

The experiment in this paper employed a WAW-2000 model computer-controlled electro-hydraulic servo universal testing machine (Shanghai Bainuo Testing Instrument Co., Ltd., Shanghai, China) with an accuracy level of 0.5. Displacement loading rates of 0.6 mm/min and 0.06 mm/min were used for the compressive strength and tensile strength tests, respectively. Three samples were randomly picked for compressive and tensile strength testing before the experiment, obtaining one group of three values each time. The median was taken as a reference, and the range was considered reasonable if the difference between the maximum, minimum and median was within 15% of the median; the average of the three measurements was taken as the strength value. The tensile strength testing method is shown in Figure 2, and is utilized to test the splitting tensile strength of concrete [33,34]. Figure 3 illustrates a fracture surface; the failure stone symmetrically distributed on both sides of the surface indicates that the sample is linearly damaged along the diameter.

#### 2.3.2. Mass Variation and Ultrasonic Velocity Testing

The same three samples were used for mass and ultrasonic velocity testing, and the average was obtained. An electronic balance (accuracy: 0.01 g) was utilized for mass testing. The samples were dried with a towel and placed in a drying oven at 50 °C for 2 h before the test.

**Figure 4:**
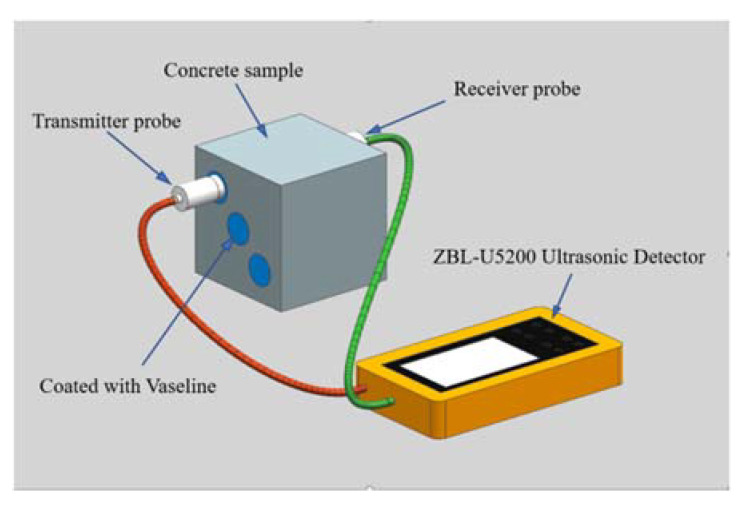
Schematic diagram of ultrasonic wave velocity test.

Ultrasonic wave velocity testing was carried out with a ZBL-U5200 Ultrasonic Detector (Beijing Zhibolian Technology Co., Ltd., Beijing, China). Its working parameters were as follows: frequency range of 1~250 kHz, sampling period of 0.4 μs, sampling length of 1024 points, acoustic time accuracy of 0.025 μs, and emission voltage of 500 V. The ultrasound opposite testing method was employed to reveal the transit time with a range of 100 mm. Three points evenly distributed on the diagonal of the surface were measured, and the average of the obtained values was used as the ultrasonic wave velocity; see Figure 4. The couplant used was Vaseline.

#### 2.3.3. Microscopic Observation

**Figure 5:**
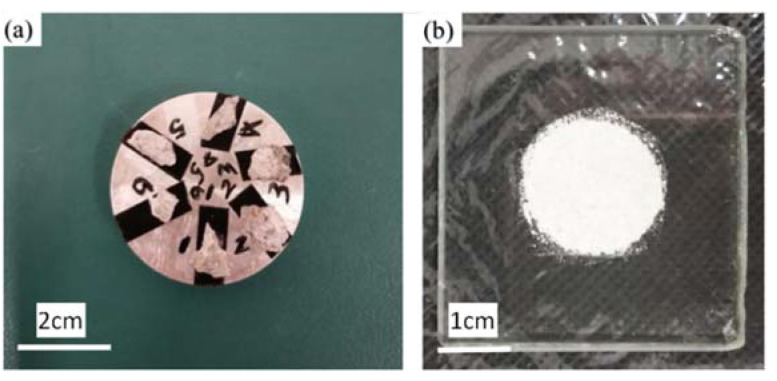
Tested samples: (**a**) example of SEM image, (**b**) example of XRD image.

The micromorphology of corrosion products was observed with a Hitachi SU70 thermal field emission scanning electron microscope (HITACHI, Tokyo, Japan), and chemical composition was analyzed using an energy dispersive spectrometer (EDS, Ametek, USA). Before testing, the corroded samples were dried and vacuum sprayed with gold (Figure 5a), thus ensuring a favorable morphology picture.

The corroded samples were X-ray diffraction (XRD) tested using a Bruker Axs D8 Advance X-ray diffractometer (Bruker, Germany) with operating parameters of 40 kV, 40 mA, 2*θ* recording range of 5–40°, step size of 0.02°, scanning time of 0.1 s/step, and CuK α-ray wavelength of λ = 0.15406 nm. For XRD detection, samples soaked for the selected period were taken out of the corrosion solution and dehydrated with anhydrous ethanol for 48 h, and then placed into a 40 °C vacuum drying oven for 48 h, followed by grinding into powder with a ceramic mortar. Finally, the powder was passed through a 80 μm sieve and tested with X-ray diffraction (Figure 5b).

## 3. Results and Discussion

In the below Figures, “3MC” and “5MC” represent concrete samples with a *w*/*c* of 0.33 and 0.5 in the compound solution of magnesium sulfate and sodium chloride (MC), “3MS” and “5MS” samples with a *w*/*c* of 0.33 and 0.5 in the magnesium sulfate solution (MS), “3SS” and “5SS” samples with a *w*/*c* of 0.33 and 0.5 in the sodium sulfate solution (SS), and “3CW” and “5CW” the samples with a *w*/*c* of 0.33 and 0.5 in clear water (CW, control solution).

### 3.1. Tensile and Compressive Strength Analysis

**Figure 6:**
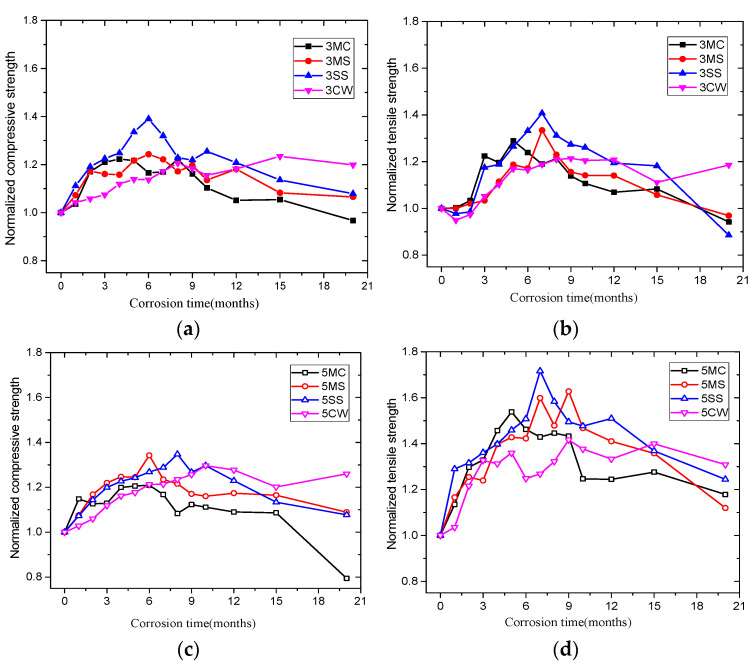
Variation in the normalized strength value of concrete over corrosion time: (**a**) compressive strength of *w*/*c* = 0.33, (**b**) tensile strength of *w*/*c* = 0.33, (**c**) compressive strength of *w*/*c* = 0.50, (**d**) tensile strength of *w*/*c* = 0.50.

The compressive and tensile strength of the high-strength and ordinary concrete samples soaked in the four corrosive solutions is shown in Appendix A: Table A1, Table A2, Table A3 and Table A4. The normalized strength ratio was obtained by dividing the tensile strength change by the initial strength (Figure 6). Figure 6 reveals that the samples’ strength underwent two stages. The first stage was an increase in compressive and tensile strength; in the second stage, strength in the control solution tended to be stable and remained basically unchanged, while that in the other three solutions decreased to varying degrees. The period ranging from the 6th to 9th month separated the two stages. In addition, the compressive strength of the high-strength concrete sample in the SS solution with low *w*/*c* climbed the most during the increase stage, which is more obvious than that of the sample with with high *w*/*c*.

Compared with CW, in addition to the increase in the strength of the concrete in the first stage due to the continuous hydration, the ions in the corrosive solutions reacted with the solution infusing the pores of the material, and the generated product filled the interior of the pores, which further increased the strength of the material. This cannot be ignored. The decline in strength in the corrosive solutions was associated with material deterioration caused by reactions between corrosive ions and this internal material, which then expanded and destroyed the internal structure.

Compared with samples in other corrosive solutions, samples in MC during the late corrosion stage saw a more significant decline in compressive strength than tensile strength. The compressive strength in MC drops the most, while the tensile strength in all corrosive solutions shared basically the same descending trend.

### 3.2. Mass Variation and Wave Velocity Analysis

**Figure 7:**
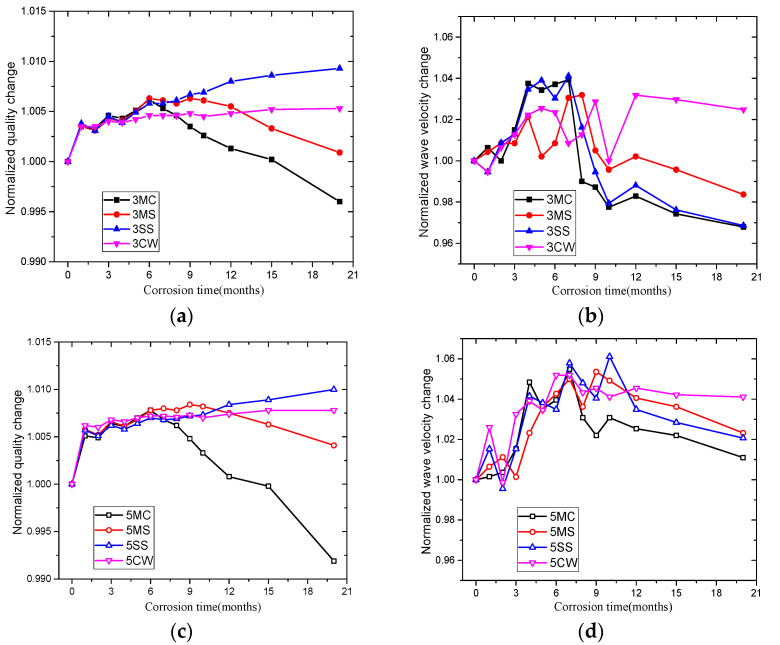
Normalized mass and ultrasonic wave velocity variation in concrete specimens in different solutions over corrosion time: (**a**) mass variation in *w*/*c* = 0.33 samples, (**b**) wave velocity variation in *w*/*c* = 0.33 samples, (**c**) mass variation in *w*/*c* = 0.50 samples, (**d**) wave velocity variation in *w*/*c* = 0.50 samples.

The mass of samples with different *w*/*c* in the four solutions during corrosion is shown in Appendix A: Table A5. The normalized mass ratio was obtained by dividing mass change by the initial mass, as shown in Figure 7a,c. The figures indicated that concrete undergoes a faster increase in mass during the first immersion month due to water absorption. The mass of samples in MC and MS mounted during the early stage (during the first six months) and dwindled in the late stage (after six months). The changes in mass after 20 months of immersion are shown in Table 4. The mass growth of samples with a large *w*/*c* was relatively higher than that of those with a small *w*/*c*, which is driven by higher internal porosity and more pore space, leading to easier entry by external corrosive ions.

The ultrasonic wave velocity of the samples is shown in Appendix A: Table A6. The normalized wave velocity ratio was obtained by dividing the change in wave velocity by the initial wave velocity, as shown in Figure 7b,d. The ultrasonic wave velocity also helps in understanding changes in samples’ internal structures after corrosion. A lower velocity indicated more pores or cracks inside the concrete and more serious corrosion, while a higher velocity indicated a denser internal structure. Figure 7 shows that despite a short wave-shaped fluctuation, the change in wave velocity correlates with the change in strength, and experiences two stages of an initial increase followed by a decrease or stabilization.

### 3.3. SEM Analysis

**Figure 8:**
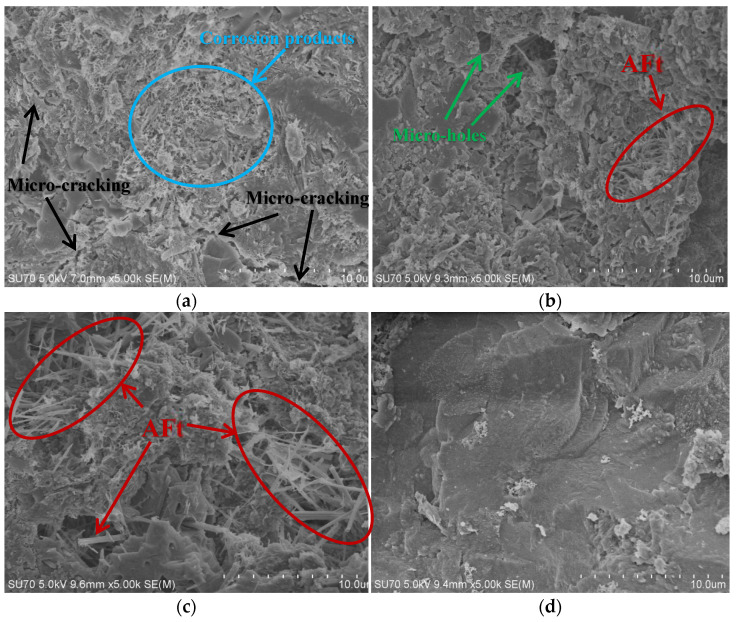
SEM images of concrete after immersion in different solutions for 10 months: (**a**) MC, (**b**) MS, (**c**) SS, (**d**) CW.

After samples were immersed in solutions for 10 months and subjected to complete compressive strength testing, several small fragments were taken from them and used for micromorphology observation using a scanning electron microscope (SEM) and for chemical composition detection using an energy dispersive spectrometer (EDS). The micromorphology is shown in Figure 8.

Figure 8a shows the presence of a large number of corrosion products and microcracks in samples immersed in MC, undermining internal strength. Figure 8b shows micro-holes and reaction products such as ettringite (abbreviated as AFt) inside concrete immersed in MS. Figure 8c finds delayed ettringite formation that appeared as needles in sample pores, dominating the corrosion products and gradually undermining internal structure as time goes by, inside samples immersed in SS. Figure 8d indicates relatively dense samples with no signs of corrosion immersed in CW.

### 3.4. XRD Analysis

**Figure 9:**
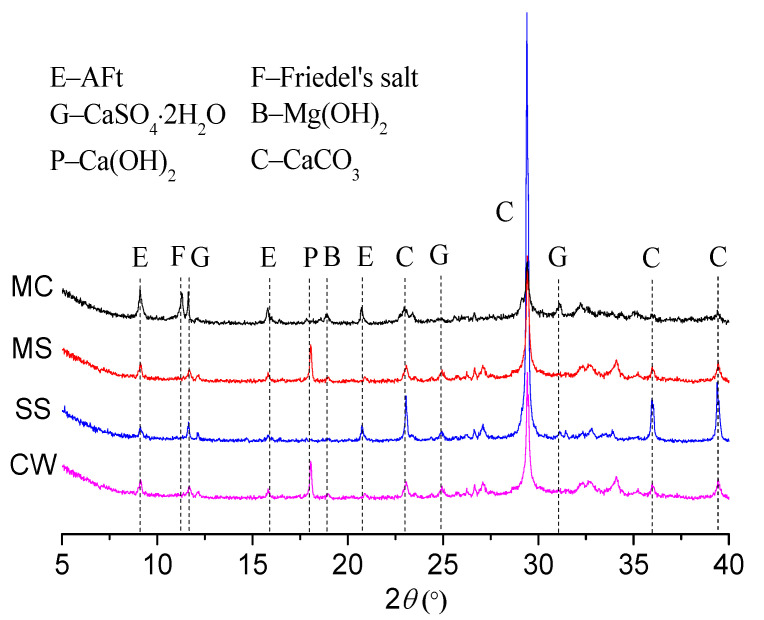
XRD patterns of cement paste in different corrosive solutions.

Samples soaked for 10 months were ground into powder and tested with an X-ray diffractometer to detect their diffraction pattern and analyze their mineral composition, as shown in Figure 9. Results found that corrosion products including ettringite, Friedel’s salt, gypsum, and magnesium hydroxide prevailed in MC; ettringite, gypsum and magnesium hydroxide dominated in MS; ettringite and gypsum reigned in SS; while in CW, more Ca(OH)_2_ could be found inside the concrete, along with small amounts of ettringite and gypsum, etc.

### 3.5. Correlation Analysis

The simplest linear correlation was employed to analyze the correlation of physical and mechanical parameters. The Pearson correlation coefficient (*R_xy_*) [35] was introduced, and the calculation could be performed using Equation (1). If |*R_xy_*| < 0.3, the two variables are not correlated; if 0.3 ≤ |*R_xy_*| < 0.5, they are weakly correlated; if 0.5 ≤ |*R_xy_*| < 0.8, they are moderately correlated; if 0.8 ≤ |*R_xy_*|, they are highly correlated.

(1) $$R_{xy}=\frac{n\sum_{i=1}^{n}x_{i}y_{i}-\left(\sum_{i=1}^{n}x_{i}\right)\left(\sum_{i=1}^{n}y_{i}\right)}{\sqrt{\left[n\sum_{i=1}^{n}x_{i}^{2}-{\left(\sum_{i=1}^{n}x_{i}\right)}^{2}\right]\left[n\sum_{i=1}^{n}y_{i}^{2}-{\left(\sum_{i=1}^{n}y_{i}\right)}^{2}\right]}}$$

**Figure 10:**
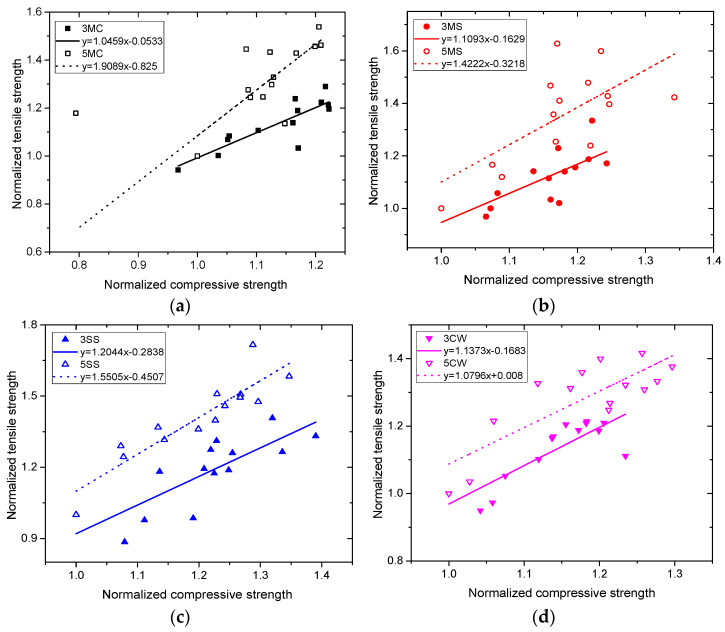
Correlations between compressive strength and tensile strength of concrete in different corrosive solutions: (**a**) MC, (**b**) MS, (**c**) SS, (**d**) CW.

Figure 10 shows the linear correlation between compressive strength and tensile strength for samples in each of the four solutions, and Table 5 reveals the resulting correlation coefficients. As can be seen from Table 5, most samples were highly correlated, except for 5MC, 3MS and 5MS, which were moderately correlated, indicating a favorable linear correlation between compressive strength and tensile strength. For linear correlation, samples with low *w*/*c* (high-strength concrete) were relatively more stable than those with high *w*/*c* (ordinary concrete).

**Figure 11:**
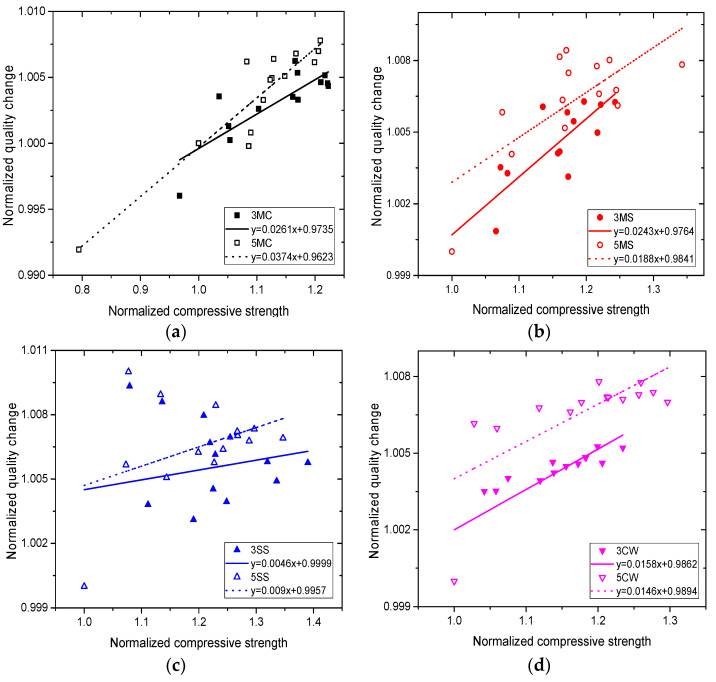
Correlations between compressive strength and mass of concrete in different corrosive solutions: (**a**) MC, (**b**) MS, (**c**) SS, (**d**) CW.

Figure 11 shows the linear correlations between compressive strength and mass, and Table 6 reveals their correlation coefficients. Results supported an extremely poor, if not unobserved, correlation in SS, which can be attributed to the mass loss of samples lagging far behind the strength decline during corrosion, although samples in SS were subject to damage from corrosion and cracking. A high correlation was observed in the other three solutions, except for 5MS and 5CW, which were moderately correlated, indicating that the strength change of samples in the three solutions could be correlated with or indicated by the mass change.

**Figure 12:**
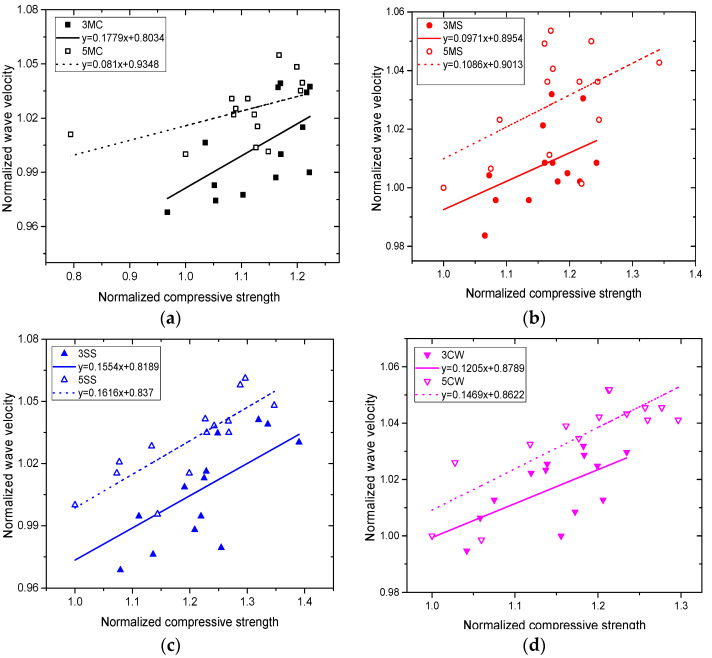
Correlations between concrete compressive strength and ultrasonic wave velocity in different corrosive solutions: (**a**) MC, (**b**) MS, (**c**) SS, (**d**) CW.

Figure 12 shows the linear correlations between compressive strength and ultrasonic wave velocity, and Table 7 reveals their correlation coefficients. It could be concluded that the correlation is less significant than the above. Except for 5SS and 5CW, in which correlations were high, the remaining samples had moderate correlations. The correlations in samples in SS and CW were better than in those in MC and MS, and the correlations in SS and CW with high *w*/*c* were better than in those with low *w*/*c*. The reasons for the lower correlation could be due to the couplant: its proximity to the concrete test surface for each test, and whether coupling layer was mixed with sand or air, etc. It could also be related to individual operation problems, such as the front of the sensor not being strictly aligned. Therefore, it is unreasonable to characterize strength in a corrosive environment by merely taking the ultrasonic wave velocity.

## 4. Conclusions

The correlations between physical and mechanical parameters were investigated based on an analysis of the macroscopic physical and mechanical properties, micromorphology and composition of concrete samples in seawater-simulating corrosive solutions. The following conclusions were drawn:

1. Using the correlation analysis method, a quantitative characterization relationship between the physical and mechanical parameters of the material was obtained, which further enriches the development of concrete durability theory. In engineering, the reliability of concrete structures in corrosive environments can be evaluated through appropriate physical parameters such as mass variation, which further enriches the detection methods for concrete structures in corrosive environments.
2. The compressive strength and tensile strength displayed a significant linear correlation with corrosion. The compressive strength of samples in solutions other than SS also significantly correlated with mass. However, the linear correlation between compressive strength and ultrasonic wave velocity was weaker than the former two.
3. The linear correlation between compressive strength and tensile strength in samples with low w/c (high-strength concrete) was relatively more stable than in those with high w/c (ordinary concrete). Compressive strength showed the largest increase under SS immersion in samples with low w/c (high-strength concrete), which was more visible than that in samples with high w/c. The increase in mass was greater for samples with high w/c than those with low w/c.

According to the findings, the monitoring of health and calculation of residual reliability for active concrete structures can be carried out using the relationship established in this paper. This method can also be generalized for other similar studies.

## Figures and Tables

**Table 1:** Concentrations of major harmful ions in seawater at selected coastal ports in China.

Ionic Species	Concentrations (mg/L)						
	Dalian	Tianjin	Qingdao	Lianyungang	Beilun	Xiamen	Zhanjiang
Cl^−^	15,900	16,842	16,000	10,700	11,760	15,440	—
${\mathrm{SO}}_{4}^{2-}$	2171	2489	2400	2289	168	2140	2198
Mg^2+^	1102	1156	1445	1159	803	1172	880

**Table 2:** Chemical composition of cement by wt. %.

CaO	SiO_2_	Al_2_O_3_	Fe_2_O_3_	SO_3_	MgO	K_2_O	TiO_2_	Na_2_O	Loss
67.18	15.92	5.55	4.19	3.18	1.71	0.91	0.59	0.32	0.45

**Table 3:** Concentration of corrosive solutions (wt.%).

Type of Solution	Symbols	Water	MgSO_4_	NaCl	Na_2_SO_4_
5% MgSO_4_ + 10% NaCl solution	MC	85	5	10	0
5% MgSO_4_ solution	MS	95	5	0	0
5% Na_2_SO_4_ solution	SS	95	0	0	5
clear water	CW	100	0	0	0

**Table 4:** Mass change ratio (wt.%) of concrete specimens under different solutions.

3MC	5MC	3MS	5MS	3SS	5SS	3CW	5CW
−0.40	−0.81	0.09	0.41	0.93	1.00	0.53	0.78

**Table 5:** Correlations between compressive strength and tensile strength.

Specimens	3MC	5MC	3MS	5MS	3SS	5SS	3CW	5CW
*R_xy_*	0.87	0.74	0.75	0.67	0.81	0.91	0.84	0.82

**Table 6:** Correlations between compressive strength and mass.

Specimens	3MC	5MC	3MS	5MS	3SS	5SS	3CW	5CW
*R_xy_*	0.85	0.93	0.85	0.72	0.20	0.39	0.84	0.70

**Table 7:** Correlations between compressive strength and ultrasonic wave velocity.

Specimens	3MC	5MC	3MS	5MS	3SS	5SS	3CW	5CW
*R_xy_*	0.62	0.50	0.51	0.50	0.67	0.82	0.67	0.83

## Data Availability

Data are contained within the article.

## Appendix A

**Table A1:** Compressive strength values of concrete with *w*/*c* = 0.33 (Unit: MPa).

Time (Months)	3MC	R^2^	3MS	R^2^	3SS	R^2^	3CW	R^2^
0	59.54	5.49	59.54	5.49	59.54	5.49	59.54	5.49
1	61.63	5.41	63.85	7.28	66.17	9.43	62.03	9.48
2	69.70	1.84	69.85	1.63	70.90	6.93	63.00	8.51
3	72.03	0.96	69.10	15.50	72.93	3.40	64.00	9.00
4	72.80	2.86	68.93	7.60	74.33	6.35	66.65	10.11
5	72.45	4.03	72.43	3.10	79.53	4.30	67.80	6.90
6	69.40	7.92	74.00	5.23	82.77	7.24	67.70	6.71
7	69.65	1.77	72.73	8.26	78.57	2.37	69.80	10.32
8	72.73	3.93	69.77	7.86	73.15	4.17	71.80	9.80
9	69.17	5.56	71.25	9.51	72.60	4.95	70.45	0.78
10	65.67	2.91	67.60	0.28	74.70	9.15	68.80	8.24
12	62.60	2.69	70.33	1.87	71.95	8.80	70.40	9.76
15	62.75	2.05	64.47	5.85	67.63	0.81	73.50	9.05
20	57.60	3.37	63.45	6.15	64.25	0.64	71.40	5.80

**Table A2:** Tensile strength values of concrete with *w*/*c* = 0.33 (Unit: MPa).

Time (Months)	3MC	R^2^	3MS	R^2^	3SS	R^2^	3CW	R^2^
0	5.72	0.06	5.72	0.06	5.72	0.06	5.72	0.06
1	5.73	0.68	5.72	0.82	5.59	0.04	5.43	0.52
2	5.91	0.74	5.83	0.51	5.63	0.50	5.56	0.80
3	7.00	0.85	5.91	0.77	6.72	0.04	6.02	0.13
4	6.84	0.67	6.37	0.04	6.79	0.37	6.30	0.19
5	7.37	0.83	6.79	0.76	7.23	0.19	6.68	0.54
6	7.08	0.70	6.70	0.02	7.61	0.84	6.66	0.21
7	6.80	0.63	7.63	0.34	8.05	0.98	6.79	0.30
8	6.94	0.47	7.03	0.34	7.50	0.85	6.91	0.29
9	6.51	0.81	6.61	0.79	7.28	0.97	6.94	0.58
10	6.33	0.81	6.53	0.55	7.21	0.31	6.89	0.38
12	6.11	0.11	6.52	0.48	6.82	0.40	6.91	0.48
15	6.19	0.67	6.05	0.59	6.76	0.79	6.35	0.92
20	5.39	0.48	5.54	0.36	5.06	0.58	6.78	0.93

**Table A3:** Compressive strength values of concrete with *w*/*c* = 0.50 (Unit: MPa).

Time (Months)	5MC	R^2^	5MS	R^2^	5SS	R^2^	5CW	R^2^
0	45.12	2.63	45.12	2.63	45.12	2.63	45.12	2.63
1	51.80	2.30	48.50	3.87	48.40	4.70	46.37	2.21
2	50.80	3.11	52.70	0.71	51.60	6.01	47.80	0.85
3	50.93	0.76	55.00	2.69	54.10	2.12	50.45	1.34
4	54.10	2.91	56.25	0.64	55.35	2.48	52.40	3.25
5	54.40	3.42	56.15	2.33	56.05	1.49	53.10	0.99
6	54.55	2.19	60.57	0.83	57.20	3.11	54.70	1.83
7	52.67	1.66	55.70	0.85	58.10	4.11	54.77	1.35
8	48.85	1.63	54.85	1.91	60.77	2.72	55.70	6.44
9	50.67	5.49	52.80	1.70	57.17	9.47	56.70	2.97
10	50.13	1.08	52.35	0.50	58.50	4.02	58.50	3.39
12	49.17	3.10	52.95	5.75	55.47	6.02	57.60	2.69
15	49.00	1.13	52.55	1.77	51.13	1.53	54.20	2.11
20	35.83	0.55	49.13	1.80	48.60	3.77	56.83	5.70

**Table A4:** Tensile strength values of concrete with *w*/*c* = 0.50 (Unit: MPa).

Time (Months)	5MC	R^2^	5MS	R^2^	5SS	R^2^	5CW	R^2^
0	4.12	0.07	4.12	0.07	4.12	0.07	4.12	0.07
1	4.67	0.19	4.80	0.45	5.31	0.59	4.26	0.64
2	5.34	0.17	5.16	0.71	5.42	0.42	5.00	0.59
3	5.47	0.55	5.10	0.18	5.60	0.86	5.46	0.81
4	6.00	0.62	5.75	0.67	5.76	0.74	5.40	0.26
5	6.33	0.40	5.88	0.44	6.01	0.16	5.60	0.90
6	6.02	0.32	5.86	0.80	6.21	0.37	5.14	0.13
7	5.88	0.66	6.58	0.93	7.07	0.20	5.22	0.11
8	5.95	0.06	6.09	0.65	6.52	0.20	5.44	0.48
9	5.90	0.74	6.70	0.60	6.15	0.14	5.83	0.63
10	5.13	0.27	6.04	0.01	6.08	0.54	5.67	0.69
12	5.12	0.29	5.81	0.66	6.21	0.29	5.49	0.81
15	5.25	0.84	5.59	0.77	5.63	0.53	5.76	0.53
20	4.85	0.26	4.61	0.98	5.12	0.42	5.39	0.81

**Table A5:** Average mass of concrete (Unit: g).

Time (Months)	3MC	5MC	3MS	5MS	3SS	5SS	3CW	5CW
0	2304.82	2287.03	2296.89	2311.85	2298.30	2334.10	2330.96	2328.35
1	2313.00	2298.68	2304.98	2325.33	2307.05	2347.31	2339.13	2342.70
2	2312.41	2298.32	2304.09	2323.81	2305.44	2345.92	2339.17	2342.26
3	2315.47	2301.65	2306.49	2327.12	2308.71	2348.68	2340.33	2344.13
4	2314.85	2301.09	2306.34	2325.98	2307.36	2347.54	2340.11	2343.76
5	2316.68	2303.00	2308.32	2327.48	2309.57	2349.00	2340.84	2344.63
6	2319.22	2304.85	2311.25	2329.95	2311.54	2350.48	2341.80	2345.11
7	2317.13	2302.58	2311.01	2330.39	2311.64	2349.91	2341.64	2345.06
8	2315.31	2301.20	2310.27	2329.79	2312.42	2350.21	2341.72	2344.90
9	2312.93	2298.03	2311.31	2331.33	2313.67	2350.93	2342.21	2345.32
10	2310.85	2294.54	2310.81	2330.70	2314.26	2351.20	2341.41	2344.65
12	2307.82	2288.88	2309.41	2329.14	2316.59	2353.77	2342.24	2345.54
15	2305.39	2286.53	2304.42	2326.52	2318.05	2354.95	2343.10	2346.51
20	2295.66	2268.59	2298.85	2321.29	2319.74	2357.44	2343.22	2346.43

**Table A6:** Average ultrasonic wave velocity of concrete (Unit: km/s).

Time (Months)	3MC	5MC	3MS	5MS	3SS	5SS	3CW	5CW
0	4.67	4.55	4.70	4.60	4.62	4.58	4.71	4.62
1	4.70	4.56	4.72	4.63	4.60	4.65	4.69	4.74
2	4.67	4.57	4.74	4.66	4.66	4.56	4.74	4.61
3	4.74	4.62	4.74	4.61	4.68	4.65	4.77	4.77
4	4.85	4.77	4.80	4.71	4.78	4.77	4.82	4.80
5	4.83	4.71	4.71	4.77	4.80	4.76	4.83	4.78
6	4.84	4.73	4.74	4.80	4.76	4.74	4.82	4.86
7	4.85	4.80	4.84	4.83	4.81	4.85	4.75	4.86
8	4.62	4.69	4.85	4.77	4.70	4.80	4.77	4.82
9	4.61	4.65	4.72	4.85	4.60	4.77	4.85	4.83
10	4.57	4.69	4.68	4.83	4.53	4.86	4.71	4.81
12	4.59	4.67	4.71	4.79	4.57	4.74	4.86	4.83
15	4.55	4.65	4.68	4.77	4.51	4.71	4.85	4.82
20	4.52	4.60	4.62	4.71	4.48	4.68	4.83	4.81

## References

[1] Kumar M.P., Mini K.M., Rangarajan M. (2018). Ultrafine GGBS and calcium nitrate as concrete admixtures for improved mechanical properties and corrosion resistance. Constr. Build. Mater..

[2] Raghav M., Karthick S., Park T., Lee H.S. (2021). Assessment of corrosion performance of steel rebar in snail shell ash blended cements under marine environments. Materials.

[3] Da B., Yu H.F., Ma H.Y., Wu Z.Y. (2018). Reinforcement corrosion research based on the linear polarization resistance method for coral aggregate seawater concrete in a marine environment. Anti-Corros. Methods M.

[4] Qiao X., Chen J.K. (2019). Correlation of propagation rate of corrosive crack in concrete under sulfate attack and growth rate of delayed ettringite. Eng. Fract. Mech..

[5] Chen J.K., Jiang M.Q., Zhu J. (2008). Damage evolution in cement mortar due to erosion of sulphate. Corros. Sci..

[6] Chu H.Y., Chen J.K. (2013). Evolution of viscosity of concrete under sulfate attack. Constr. Build. Mater..

[7] Monticelli C., Natali M.E., Balbo A., Chiavari C., Zanotto F., Manzi S., Bignozzi M.C. (2016). A study on the corrosion of reinforcing bars in alkali-activated fly ash mortars under wet and dry exposures to chloride solutions. Cem. Concr. Res..

[8] Skaropoulou A., Tsivilis S., Kakali G., Sharp J.H., Swamy R.N. (2009). Long term behavior of Portland limestone cement mortars exposed to magnesium sulfate attack. Cem. Concr. Compos..

[9] Wang D.Z., Zhou X.M., Meng Y.F., Chen Z. (2017). Durability of concrete containing fly ash and silica fume against combined freezing-thawing and sulfate attack. Constr. Build. Mater..

[10] Jaworska-Wędzińska M., Jasińska I. (2022). Durability of mortars with fly ash subject to freezing and thawing cycles and sulfate attack. Materials.

[11] Yu H.F., Tan Y.S., Feng T.T. (2019). Study of temporal change in the chloride diffusion coefficient of concrete. ACI Mater. J..

[12] Da B., Yu H.F., Ma H.Y., Tan Y.S., Mi R.J., Dou X.M. (2016). Chloride diffusion study of coral concrete in a marine environment. Constr. Build. Mater..

[13] Fu J.B. (2005). Several Key Techniques about Construction Material Resourcialization of Sea Sand.

[14] Dehwah H.A.F. (2007). Effect of sulfate concentration and associated cation type on concrete deterioration and morphological changes in cement hydrates. Constr. Build. Mater..

[15] Jiang L., Niu D.T. (2016). Study of deterioration of concrete exposed to different types of sulfate solutions under drying-wetting cycles. Constr. Build. Mater..

[16] Jin Z.Q., Sun W., Zhang Y.S., Jiang J.Y., Lai J.Z. (2007). Interaction between sulfate and chloride solution attack of concretes with and without fly ash. Cem. Concr. Res..

[17] Haufe J., Vollpracht A. (2019). Tensile strength of concrete exposed to sulfate attack. Cem. Concr. Res..

[18] Santhanam M., Cohen M., Olek J. (2006). Differentiating seawater and groundwater sulfate attack in Portland cement mortars. Cem. Concr. Res..

[19] Wang K., Nelsen D.E., Nixon W.A. (2006). Damaging effects of deicing chemicals on concrete materials. Cem. Concr. Compos..

[20] Li L.L., Shi J.P., Kou J.L. (2021). Experimental study on mechanical properties of high-ductility concrete against combined sulfate attack and dry-wet cycles. Materials.

[21] De Weerdt K., Orsáková D., Geiker M.R. (2014). The impact of sulphate and magnesium on chloride binding in Portland cement paste. Cem. Concr. Res..

[22] Maes M., De Belie N. (2014). Resistance of concrete and mortar against combined attack of chloride and sodium sulphate. Cem. Concr. Compos..

[23] Benli A., Karatas M., Gurses E. (2017). Effect of sea water and MgSO_4_ solution on the mechanical properties and durability of self-compacting mortars with fly ash/silica fume. Constr. Build. Mater..

[24] Zhang M.H., Chen J.K., Lv Y.F., Wang D.J., Ye J. (2013). Study on the expansion of concrete under attack of sulfate and sulfate-chloride ions. Constr. Build. Mater..

[25] Cheng S.K., Shui Z.H., Sun T., Gao X., Guo C. (2019). Synergistic effects of sulfate and magnesium ions on chloride diffusion behaviors of Portland cement mortar. Constr. Build. Mater..

[26] Xu H., Chen J.K. (2020). Coupling effect of corrosion damage on chloride ions diffusion in cement based materials. Constr. Build. Mater..

[27] Sun C., Chen J.K., Zhu J., Zhang M.H., Ye J. (2013). A new diffusion model of sulfate ions in concrete. Constr. Build. Mater..

[28] Harbulakova V.O., Estokova A., Kovalcikova M. (2017). Correlation analysis between different types of corrosion of concrete containing sulfate resisting cement. Environment.

[29] Barbudo A., Galvín A.P., Agrela F., Ayuso J., Jiménez J.R. (2012). Correlation analysis between sulphate content and leaching of sulphates in recycled aggregates from construction and demolition wastes. Waste Manag..

[30] (2009). Method of Testing Cements-Determination of Strength.

[31] (2015). Standard Test Method for Length Change of Hydraulic-Cement Mortars Exposed to a Sulfate Solution.

[32] (2009). Standard for Test Methods of Long-Term Performance and Durability of Ordinary Concrete.

[33] (2011). Standard Test Method for Splitting Tensile Strength of Cylindrical Concrete Specimens.

[34] (2019). Standard for Test Methods of Concrete Physical and Mechanical Properties.

[35] Kreyszig E. (2011). Advanced Engineering Mathematics.

